# Depression and health-related quality of life in cardiovascular patients: A cross-sectional study on the mediating roles of fear, attention, and avoidance

**DOI:** 10.1097/MD.0000000000050147

**Published:** 2026-08-07

**Authors:** Hwan-Cheol Park, Jihyun Oh

**Affiliations:** a Division of Cardiology, Department of Internal Medicine, Hanyang University Guri Hospital, Guri City, South Korea; b Department of Nursing, College of Nursing and Health, Kongju National University, Kongju, South Korea.

**Keywords:** anxiety, attention, avoidance, depression, fear, HRQoL

## Abstract

Depression and anxiety are prevalent among patients with cardiovascular disease (CVD) and detrimentally affect their health-related quality of life (HRQoL). Although current guidelines recommend screening for both conditions, the mechanisms underlying the association between depression and HRQoL, particularly through the anxiety subdomains of fear, attention, and avoidance, remain unclear. This study investigates how anxiety-related factors mediate the relationship between depression and HRQoL in patients with CVD. This cross-sectional study analyzed 204 CVD outpatients from a tertiary hospital in South Korea. Each patient completed the Patient Health Questionnaire-9 to assess depression, the Cardiac Anxiety Questionnaire to evaluate anxiety subdomains, and the 12-Item Short Form Health Survey to measure HRQoL. Serial multiple mediation analyses were conducted to examine the direct and indirect associations between depression and HRQoL. This approach allowed the potential pathways of mediators to be simultaneously tested, particularly the sequential path from fear to attention to avoidance. Higher depression scores showed a significant negative association with HRQoL (total effect: β = -0.5449, *P* < .001). Moreover, indirect pathways were observed: increased depression was associated with heightened levels of fear, attention, and avoidance. The direct association between depression and HRQoL remained significant when the indirect pathways were controlled (β = -0.3888, *P* < .001), supporting a partial mediation model. The overall mediation model explained 18% of the variance in HRQoL. Our findings suggest that a complex interplay between depression and anxiety subdomains influences quality of life among patients with CVD. Therefore, future prospective or interventional studies are needed to evaluate whether interventions targeting depressive symptoms and maladaptive anxiety responses can improve HRQoL in this population.

## 1. Introduction

Nearly half of patients with cardiovascular disease (CVD) experience increased levels of depression and anxiety.^[1]^ Furthermore, depression can be a risk factor for adverse cardiovascular outcomes in patients with CVD.^[2,3]^ Although the link between depression and CVD outcomes is well-established, the underlying psychological pathways – specifically how anxiety subcomponents bridge depression and health-related quality of life (HRQoL) – remain a critical knowledge gap.^[4,5]^

Evidence about the relationships among depression, anxiety, and HRQoL has been inconsistent.^[6–8]^ Whereas some studies suggest a strong correlation,^[9,10]^ others report no significant associations.^[11,12]^ Crucially, although previous studies have shown evidence of associations,^[6–10]^ they have primarily established simple relationships without elucidating the distinct pathways through which specific anxiety traits influence outcomes. This study uses the Cardiac Anxiety Questionnaire (CAQ) to analyze anxiety through its three dimensions—fear, attention, and avoidance. Rather than merely confirming a relationship between anxiety and health outcomes, it examines the underlying mechanisms by which these dimensions mediate the effects of depression on HRQoL.

In this study, cardiac anxiety is conceptualized as a broad construct comprising three subdomains – fear, attention, and avoidance. This approach clarifies not only whether anxiety is associated with HRQoL, but also which specific anxiety-related responses explain this pathway. Previous studies have not fully elucidated the relationships among HRQoL, depression, and anxiety in patients with CVD, and few studies have explored whether anxiety mediates the relationship between depression and HRQoL in patients with CVD. Although current CVD prevention guidelines recommend screening for both anxiety and depression to identify the individuals at greatest risk,^[13]^ the role of anxiety as a risk factor for CVD has not been established as definitively as that of depression.^[14]^ Suls and Bunde^[15]^ pointed out that the relationship between anxiety and CVD remains inconclusive, highlighting the complexities of differentiating the effects of specific negative affective states on cardiovascular risk. Since then, researchers have increasingly emphasized the importance of clarifying the role of anxiety in cardiovascular outcomes beyond its association with depression.^[16]^ However, few studies have examined how anxiety – particularly its specific components of fear, attention, and avoidance – might function as a mediator in the relationship between depression and HRQoL in patients with CVD. Therefore, it is necessary to analyze both depression and anxiety to determine whether and how they directly and indirectly affect the HRQoL of patients with CVD.

According to the World Health Organization, HRQoL is a significant health outcome for all diseases,^[17]^ and patients with CVD often also experience diabetes or hypertension.^[18]^ Moreover, HRQoL in patients with CVD is significantly influenced by psychological factors, such as depression and anxiety.^[19,20]^ The psychological issues stem not only from the disease itself but also from the stress of managing symptoms, dealing with comorbidities, and undergoing various treatments.^[21]^ Previous studies on patients with CVD have shown that the subscales of the CAQ, fear, attention, and avoidance, correlate positively with depression.^[22,23]^ On the other hand, Schmitz et al^[23]^ found that CAQ fear scores had no correlation with physical QoL but were negatively correlated with mental QoL. In contrast, Hoyer et al^[22]^ reported that as the level of cardiac anxiety increased, both physical and mental QoL decreased. Those findings suggest that the relationship between anxiety and HRQoL in patients with CVD requires further investigation. More specifically, those inconsistent findings suggest that examining cardiac anxiety as a single broad construct might be insufficient and that its subdomains should be considered separately.

Nurses are often on the front line in managing patients with CVD, and they play a pivotal role in both assessment and intervention. Therefore, investigating the relationships among depression, anxiety, and HRQoL in patients with CVD is crucial for improving nursing practice. In particular, identifying the specific roles of the anxiety subcomponents could provide a nuanced understanding of how psychological factors influence HRQoL.^[24]^ Consequently, in this study, we examine the roles of fear, attention, and avoidance in the relationship between depression and HRQoL among patients with CVD. By focusing on those subcomponents, we address the existing gap in the literature regarding the mediating mechanisms linking depression and HRQoL. Because fear, attention, and avoidance might represent different emotional, cognitive, and behavioral responses to cardiac-related symptoms, identifying their specific mediating roles could help nurses develop targeted interventions for patients with CVD. For instance, if specific anxiety components are found to be strong mediators, behavioral activation interventions and meaning-centered counseling could be developed to help patients effectively integrate their illness experiences and enhance their coping strategies.

This study tested the following hypotheses:

Hypothesis 1. Depression is negatively correlated with HRQoL in patients with CVD.

Hypothesis 2. Fear mediates the relationship between depression and HRQoL in patients with CVD.

Hypothesis 3. Attention mediates the relationship between depression and HRQOL in patients with CVD.

Hypothesis 4. Avoidance mediates the relationship between depression and HRQoL in patients with CVD.

## 2. Methods

### 2.1. Study design and participants

This cross-sectional study was conducted between February 23, 2022, and July 30, 2022, and analyzed patients who were diagnosed with CVD by a cardiologist and visited the cardiovascular outpatient clinic at Hanyang University Hospital in Guri City. Patient data were collected after we explained the purpose and methods of the study. Only participants who provided consent were given the online survey link through which the data were collected. We initially recruited 216 patients with CVD. After excluding those with incomplete responses and those who withdrew from the study, 204 participants were included in the final data analysis, resulting in a response rate of 94.4%.

G*Power 3.1 was used to confirm the appropriateness of the sample size for this study. The calculation was based on a significance level of 0.05, power of 0.95, and medium effect size of 0.15, resulting in a minimum required sample size of 199 participants. Considering that the study participants were patients with CVD visiting an outpatient clinic, a dropout rate of approximately 15% was anticipated, and the questionnaires were distributed to 216 participants. Ultimately, 204 participants were included in the final statistical analysis, which is an appropriate sample size. Cases with incomplete responses (n = 12, 5.6%) were excluded from the analysis using listwise deletion. The proportion of excluded cases was small relative to the total sample, suggesting that the missing data were likely missing at random and would have a minimal effect on the overall findings and statistical power.

This study included participants who were adults (20 years and older) diagnosed with CVD by a cardiovascular specialist and who underwent follow-up assessment in the outpatient clinic. None of the participants had been diagnosed with depression or anxiety by a psychiatrist, and none were taking any related medications. A Mini-Cog screening test was conducted for patients aged ≥ 65 years to ensure the absence of cognitive impairment. We focused on patients with New York Heart Association cardiac function classifications of II–IV. Notably, participants with a prior diagnosis of depression or anxiety and those receiving related psychiatric treatment were excluded. This exclusion criterion was strategically applied to ensure a homogeneous sample and thus enable a focused investigation into the mechanisms of subclinical depressive symptoms and cardiac anxiety within a nonclinically diagnosed population. The exclusion criteria also included difficulty reading or writing in Korean.

### 2.2. Measurements

#### 2.2.1. Cardiac anxiety

Anxiety was assessed using the CAQ, which was originally developed by Eifert^[25]^ and later translated into Korean by Shin et al.^[26]^ This instrument comprises 18 items organized into three sub-domains: eight items in the fear domain, five items in the attention domain, and five items in the avoidance domain. The tool uses a 5-point Likert scale ranging from never (0) to always (4). The total score ranges from 0 to 72, with higher scores indicating a greater number of symptoms, a higher frequency of symptoms, or both. During its development, Cronbach’s α was reported to be.83, and in the present study, it was.90.

#### 2.2.2. Health-related quality of life

HRQoL was measured using the 12-Item Short Form Health Survey (SF-12),^[27]^ which consists of two major components: the Physical Component Summary Scale (PCS) and the Mental Component Summary Scale (MCS). The PCS comprises four subdomains: physical functioning, role-physical, body pain, and general health. The MCS also comprises four subdomains: mental health, role emotion, social functioning, and vitality. The SF-12 has 12 questions answered on a Likert scale, and the answers are combined, resulting in a score ranging from 0 to 100, with higher scores indicating higher HRQoL both overall and in the two component scores. In this study, the Cronbach’s α was.90, indicating a high level of reliability.

#### 2.2.3. Depression

The Patient Health Questionnaire-9 (PHQ-9)^[28]^ is a self-administered questionnaire with nine questions designed to assess depression (presence and severity) in primary care settings. This study used the Korean version of the PHQ-9 adapted from Park et al.^[29]^ The results were used to diagnose depression based on criteria in the Diagnostic and Statistical Manual of Mental Disorders, Fourth Edition (DSM-IV). The PHQ-9 aligns with the DSM-IV guidelines in gauging how frequently patients experience symptoms of depression during a 2-week period. The scoring system ranges from 0 (never) to 3 (almost every day), for a total score ranging from 0 to 27. Cumulative scores on the PHQ-9 allow for the categorization of depressive symptoms as none or minimal (0–4), mild (5–9), moderate (10–14), moderately severe (15–19), or severe (≥20). The Cronbach’s alpha was 0.85 in the selected Korean version of the PHQ-9, and it was 0.86 in our study, indicating a high level of reliability.

#### 2.2.4. Physical activity

Physical activity levels were assessed using the officially recognized Korean version of the short form of the Self-Administered International Physical Activity Questionnaire (IPAQ). The IPAQ comprehensively evaluates all forms of physical activity during leisure, indoor, outdoor, work-related, and transportation-related activities. The IPAQ classifies physical activity into three categories: inactive, minimally active, and health-enhancing. Minimally active participants met any one of the following criteria: vigorous activity for at least 20 min on ≥ 3 days per week; moderate activity (or walking) for at least 30 min on ≥ 5 days per week; or any combination of walking, moderate, or vigorous activities performed at least 5 days per week, accumulating a minimum of 600 MET min per week. Health-enhancing physical activity is the most desirable level of activity and is defined as either engaging in vigorous activity for at least 3 days per week, with a total of at least 1500 MET min per week, or performing a combination of walking, moderate, and vigorous activities 7 days per week, accumulating at least 3000 MET min per week. Any physical activity that did not meet the above criteria fell into the inactive category.

#### 2.2.5. Data collection

The participants were outpatients who visited the cardiology department, where they were completed each measure once, which took 15–20 min to complete. The participants could stop at any time without penalty. In total, 216 questionnaires were distributed to patients with CVD. Twelve questionnaires were excluded due to participant withdrawal during the survey or incomplete responses, so 204 questionnaires were included in the final analysis. An online questionnaire was also distributed to patients with CVD who agreed to participate.

### 2.3. Ethical considerations

This study was approved by the Institutional Review Board (IRB No. 2021-09-010-007) of Hanyang University Hospital, Guri-si, Gyeonggi-do. The study procedures were conducted in full compliance with internationally recognized ethical guidelines, including the Declaration of Helsinki. Furthermore, this study was conducted and reported in accordance with the Strengthening the Reporting of Observational Studies in Epidemiology guidelines. Informed consent, including consent to publish, was obtained from all participants prior to their participation. All participants were told the purpose, methods, and procedures of the study and promised that their data would not be used for any purpose other than research, and only those who agreed were allowed to participate. The online questionnaire was also distributed after the participants provided consent.

### 2.4. Statistical analyses

All statistical analyses were performed using SPSS statistical software (version 26; IBM Corp, Armonk, New York, NY, USA) and SPSS Macro PROCESS (Procedure for Causal Effect Statistical Segmentation) v3.1. A frequency analysis was used to examine the general characteristics, descriptive statistical analyses were used to calculate the means and standard deviations of the variables, and Pearson’s correlation coefficients were used to analyze correlations among the primary variables. Before conducting the mediation analysis, we assessed potential multicollinearity among the CAQ subscales using tolerance and variance inflation factor (VIF) values. Tolerance values greater than.10 and VIF values <10 were considered to indicate the absence of serious multicollinearity. Because all variables were measured using self-report questionnaires at a single time point, we also assessed the potential risk of common method bias. Harman’s single-factor test was performed using unrotated principal component analysis, including all items from the study variables. The first factor accounted for 31.1% of the total variance, which is below the recommended threshold of 50%. This finding suggests that common method bias was unlikely to substantially affect the study findings.

To examine the sequential mediating effects of the cardiac anxiety subscales, PROCESS macro model 6 was used. The sequence of mediators was specified based on the following progression of psychological stages: emotional responses to perceived threats (fear) are followed by increased attentional focus on those stimuli (attention), ultimately leading to behavioral avoidance. This directional pathway allowed for a nuanced understanding of the progressive mechanisms through which depression influences HRQoL.

**Figure 1.**
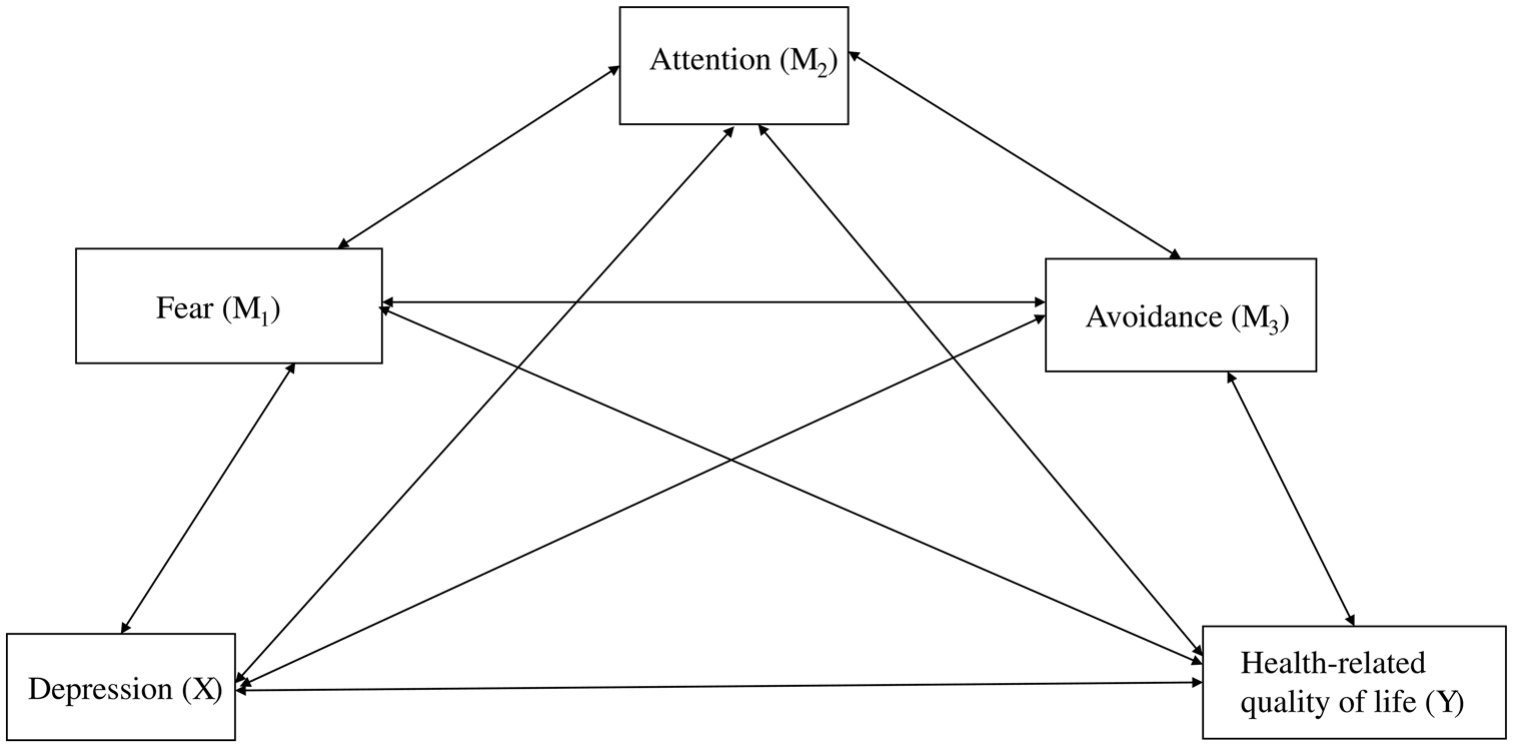
Proposed serial multiple mediation model.

The serial multiple mediation model has three mediators: the independent variable (X) is assumed to affect the first parameter (M1), which in turn affects the second parameter (M2), which then affects the third parameter (M3), thereby establishing sequential causal relationships among the parameters within a single model. The completed mediation model aimed to reveal the direct and indirect effects of the independent (X) and dependent variables (Y).^[30]^ The model used in this study is illustrated in Figure 1.

The PROCESS macro tested the mediation effects by applying the bootstrap method, extracting 10,000 samples, and analyzing the 95% bias-corrected confidence interval (BC CI). A serial multiple mediation model involves the inclusion of two or more mediators, allowing the examination of both direct and indirect effects, as well as the verification of causal relationships among the mediators. The mediating effect was considered significant if the 95% BC CI for the indirect path coefficient did not include zero. To address potential confounding, we identified variables known to influence HRQoL (age, sex, marital status, education, economic status, and clinical comorbidities). Preliminary analyses were conducted to assess their relationships with study variables. We adopted a parsimonious modeling approach to prioritize a theoretically driven model, ensure the clarity of indirect effects, and prevent over-parameterization. However, to examine the robustness of our findings, we conducted additional sensitivity analyses by adjusting for the key potential confounders of age, sex, and clinical comorbidities. The results of the adjusted models were compared with those of the primary mediation model to determine whether the direction and significance of the indirect effects remained consistent.

## 3. Results

### 3.1. General characteristics of participants

Table 1 presents the general characteristics of the participants. The mean age of the participants was 56.7 years (SD = 10.90). Of the 204 participants, 63.20% were female, and 68.60% were married. Furthermore, only 46.10% of participants reported consuming alcohol, indicating that the majority were nondrinkers. Additionally, 75.00% were nonsmokers, 56.40% engaged in regular exercise, and 51.00% reported having no religious affiliation. Among the participants, 41.70% were high school graduates, and when asked about their perceived economic status, 69.60% rated it as “middle.” Most participants (92.60 %) reported that they were currently taking medication for CVD. When asked whether their work was physically demanding, 27.00% responded that it was severely demanding, 35.30% indicated that it was moderately demanding, and 37.70% indicated that it was mildly demanding. Most of the patients (70.10%) were employed.

**Table 1:** Characteristics of the study population (N = 204).

Variable	N (%)	Mean (SD)
Mean age (years) (SD, range)		56.70 (10.90), (30–85)
Sex		
Female	129 (63.20)	
Male	75 (36.80)	
Marital status		
Single	19 (9.30)	
Married	140 (68.60)	
Divorced or widowed	45 (22.10)	
Alcohol consumption		
Yes	94 (46.10)	
No	110 (53.90)	
Smoking		
Yes	51 (25.00)	
No	153 (75.00)	
Regular exercise		
Yes	115 (56.40)	
No	89 (43.60)	
Religion		
Christianity	51 (25.00)	
Catholicism	8 (3.90)	
Buddhism	41 (20.10)	
No religion	104 (51.00)	
Education level		
Elementary	26 (12.70)	
Middle school	26 (12.70)	
High school	85 (41.70)	
≥ College	67 (32.80)	
Economic status		
High	11 (5.40)	
Middle	142 (69.60)	
Low	51 (25.00)	
Medication		
Yes	189 (92.60)	
No	15 (7.40)	
Physical burden		
Mild	77 (37.70)	
Moderate	72 (35.30)	
Severe	55 (27.00)	
Job		
Yes	143 (70.10)	
No	61 (29.90)	
Level of physical activity		
Health-enhancing physical activity	18 (8.80)	
Minimally active	76 (37.30)	
Inactive	110 (53.90)	
†Types of cardiovascular disease and physical disease		
Coronary artery disease	178 (87.30)	
Diabetes mellitus	43 (23.50)	
Hypertension	78 (38.20)	
Cerebrovascular disease	7 (3.40)	
Cancer	4 (2.00)	
Aneurysm	9 (2.00)	
Other	15 (7.40)	

SD = Standard deviation.

† Multiple responses.

Most people in our cohort – 110 (53.90%) individuals – were categorized as inactive, indicating that they did not meet the IPAQ criteria for minimal or health-enhancing physical activity. Seventy-six (37.30%) participants were classified as minimally active and met at least one minimal activity criterion. Only 18 (8.80%) participants reported health-enhancing physical activity levels. Within the study cohort, coronary artery disease was the most prevalent condition, affecting 178 (87.30%) participants. Diabetes mellitus and hypertension were reported by 43 (23.50%) and 78 (38.20%) participants, respectively. Cerebrovascular disease was present in seven (3.40%) participants, cancer in four (2.00%), and aneurysm in nine (2.00%). Additionally, 15 (7.40%) participants were diagnosed with other chronic conditions.

### 3.2. Depression, cardiac anxiety, and HRQoL

The means and standard deviations of the main variables (depression, cardiac anxiety, and HRQoL) are presented in Table 2. On the 100-point scale of the SF-12, the overall mean HRQoL score was 69.72 (8.87). The average PCS score was 67.38 (10.94), and the average MCS score was 72.07 (10.19). The mean depression score was 5.56 (4.95). The average score for cardiac anxiety was 18.73 (15.29). Among the subdomains of cardiac anxiety, the mean scores were 5.02 (5.45) for avoidance, 9.46 (7.84) for fear, and 4.24 (4.82) for attention.

**Table 2:** Levels of depression, cardiac anxiety, and quality of life (N = 204).

Variable	Min-Max	Mean (SD)
SF-12	34.70–90.30	69.72 (8.87)
PCS	38.40–92.30	67.38 (10.94)
MCS	23.30–100	72.07 (10.19)
CAQ Total	0–59	18.73 (15.29)
CAQ-Avoidance	0–20	5.02 (5.45)
CAQ-Fear	0–32	9.46 (7.84)
CAQ-Attention	0–20	4.24 (4.82)
Depression	0–22	5.56 (4.95)

CAQ = Cardiac Anxiety Questionnaire.

### 3.3. Correlations among variables

Table 3 shows the correlations among depression, cardiac anxiety, and HRQoL in patients with CVD. Depression correlated positively with total cardiac anxiety (CAQ total; r = .315, *P* < .001), and its subdomains: avoidance (r = .306, *P* < .001), fear (r = .282, *P* < .001), and attention (r = .194, *P* = .005). The total cardiac anxiety score correlated significantly positively with the CAQ-Avoidance (r = .800, *P* < .001), CAQ-Fear (r = .884, *P* < .001), and CAQ-Attention (r = .829, *P* < .001) scores, indicating high internal convergence among the subscales. In contrast, depression correlated significantly negatively with physical health (PCS; r = –.203, *P* = .004), mental health (MCS; r = –.312, *P* < .001), and overall HRQoL (SF-12; r = –.304, *P* < .001). Similarly, total cardiac anxiety was negatively associated with the PCS (r = –.291, *P* < .001), MCS (r = –.247, *P* < .001), and overall HRQoL (r = –.321, *P* < .001). Notably, CAQ-Avoidance exhibited the strongest negative association with overall HRQoL (r = –.372, *P* < .001), followed by the PCS (r = –.313, *P* < .001) and MCS (r = –.313, *P* < .001). These findings suggest that higher levels of cardiac-related anxiety and depressive symptoms are associated with poorer physical and mental HRQoL.

**Table 3:** Correlations among variables (N = 204).

Variables	Depression	CAQ Total	CAQ-Avoidance	CAQ-Fear	CAQ-Attention	PCS	MCS	SF-12
Depression	–							
CAQ Total	.315 (<.001)	–						
CAQ-Avoidance	.306 (<.001)	.800 (<.001)	–					
CAQ-Fear	.282 (<.001)	.884 (<.001)	.510 (<.001)	–				
CAQ-Attention	.194 (.005)	.829 (<.001)	.577 (<.001)	.600 (<.001)	–			
PCS	−.203 (.004)	−.291 (<.001)	−.313 (<.001)	−.183 (.009)	−.272 (<.001)	–		
MCS	−.312 (<.001)	−.247 (<.001)	−.313 (<.001)	−.153 (.029)	−.180 (.010)	.410 (<.001)	–	
SF-12	−.304 (<.001)	−.321 (<.001)	−.372 (<.001)	−.201 (.004)	−.271 (<.001)	.852 (<.001)	.827 (<.001)	–

CAQ = Cardiac Anxiety Questionnaire, MCS = Mental Component Summary Scale, PCS = Physical Components Summary Scale, SF-12 = Short Form Health Survey.

### 3.4. Examination of the relationships among fear, attention, avoidance, and depression

**Figure 2.**
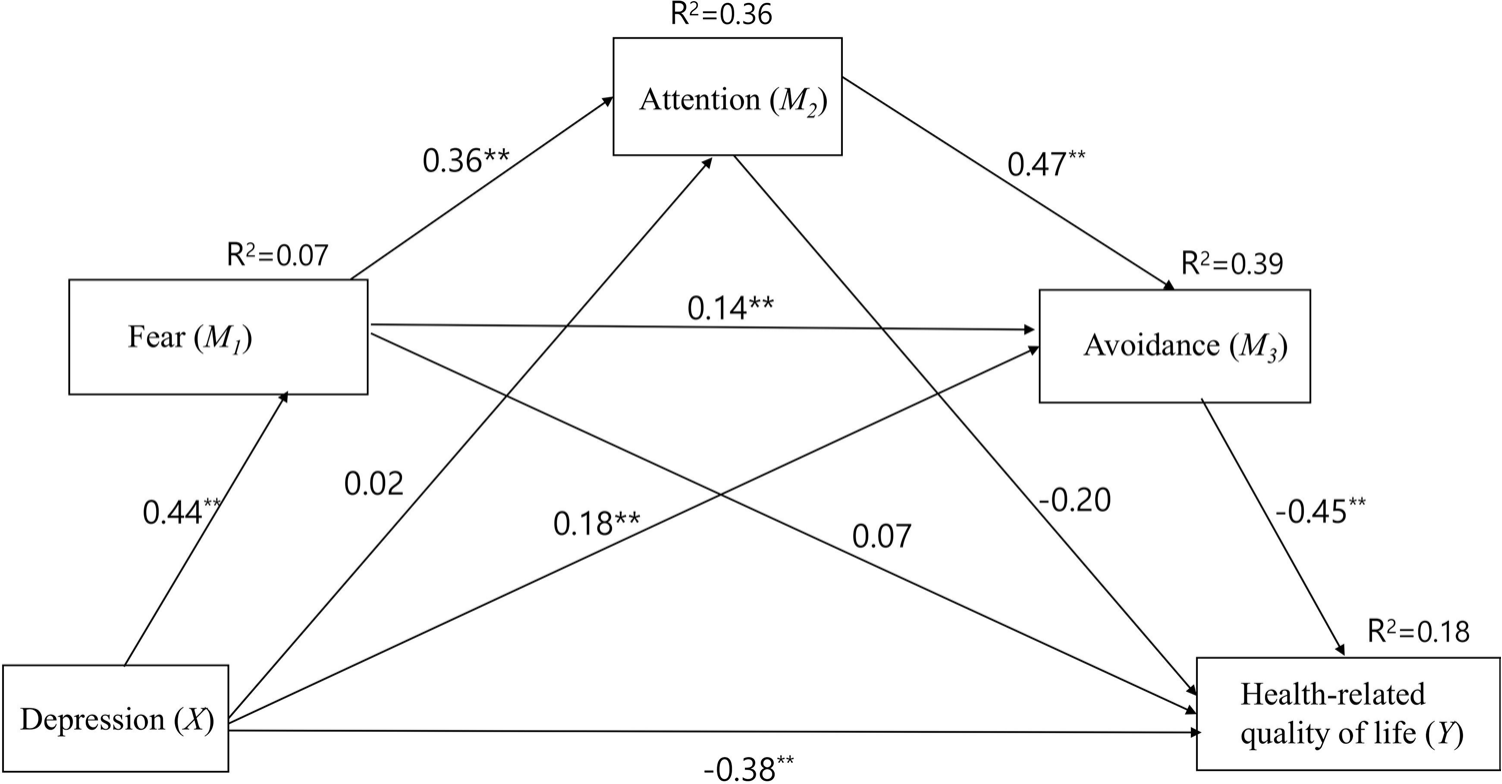
Serial multiple mediation model for the relationship between depression and HRQoL.

Before we tested the mediation model, we examined multicollinearity among the CAQ subscales. The tolerance values for the CAQ subscales ranged from.539 to.600, and the VIF values ranged from 1.666 to 1.855, indicating that multicollinearity among fear, attention, and avoidance was not a serious concern. Table 4 presents the total, direct, and indirect associations between depression and the HRQoL of CVD patients, with fear, attention, and avoidance serving as mediators. Figure 2 presents the corresponding path coefficients of those variables. The total association between depression and HRQoL was significant (β = −.5449, *P* < .001, 95% BC CI [−.7818, −.3080]). Even after accounting for the mediating variables (fear, attention, and avoidance), the direct association between depression and HRQoL remained significant (β = −.3888, *P* = .0017, 95% BC CI [−.6295, −.1481]), indicating that the relationship is partially mediated. The total indirect association through the mediators was also significant (β = −.1561, 95% BC CI [−.3001, −.0308]). Regarding the specific indirect pathways, the results revealed three significant routes: first, simple mediation through avoidance was significant (Indirect 3: β = −.0819, 95% BC CI [−.1866, −.0019]); second, sequential mediation involving fear and avoidance was also significant (Indirect 5: β = −.0297, 95% BC CI [−.0693, −.0045]); and third, the combined sequential path through fear, attention, and avoidance was also significant (Indirect 7: β = −.0346, 95% BC CI [−.0735, −.0094]). These findings clearly indicate that avoidance, whether acting independently or in sequence with fear and attention, could serve as a psychological mechanism in the relationship between depression and HRQoL. Sensitivity analyses adjusting for age, sex, and clinical comorbidities showed that the indirect relationships remained consistent in direction and significance with those observed in the primary mediation model. Those findings support the robustness of the mediation results after accounting for key potential confounders (Table S1, Supplemental Digital Content 1).

**Table 4:** Total, direct, and indirect effects in the multiple mediator model (N = 204).

Model	Effect	SE	*t*	*p*	95% BC CI
Total effect of depression on quality of life	−.544	.120	−4.535	<.001	[−.781, −.308]
Direct effect of depression on quality of life	−.388	.122	−3.184	.001	[−.629, −.148]
Total indirect effect	−.156	.069			[−.300, −.030]
Indirect effect via					
Indirect 1	.033	.049			[−.057,.141]
Indirect 2	−.005	.015			[−.042,.026]
Indirect 3	−.081	.048			[−.186, −.001]
Indirect 4	−.032	.032			[−.107,.019]
Indirect 5	−.029	.017			[−.069, −.004]
Indirect 6	−.005	.013			[−.034,.018]
Indirect 7	−.034	.016			[−.073–.009]
Indirect effect key:					
Indirect 1: Depression→Fear→Quality of life					
Indirect2: Depression→Attention→Quality of life					
Indirect3: Depression→Avoidance→Quality of life					
Indirect4: Depression→Fear→Attention→Quality of life					
Indirect5: Depression→Fear→Avoidance→Quality of life					
Indirect6: Depression→Attention→Avoidance→Quality of life					
Indirect7: Depression→Fear→ Attentions→Avoidance→Quality of life					

BC CI = bias-corrected confidence interval.

Because both the direct and indirect associations were statistically significant, these results demonstrate that the relationship between depression and HRQoL is partially mediated, rather than fully mediated, by the anxiety subdomains. Bootstrap test results indicated that avoidance mediated the relationship between depression and HRQoL, suggesting that higher depression is associated with greater avoidance, which contributes to lower HRQoL. Additionally, the combined serial mediation of fear and avoidance was significant, implying that increased depression was associated with heightened fear and avoidance that ultimately reduced HRQoL.

Overall, the proposed mediation model explained approximately 18% of the variance in HRQoL (R^2^ = .18), suggesting that the model accounted for a meaningful but relatively small proportion of HRQoL variance. Therefore, the explanatory power of the model should be interpreted cautiously; most of the variance in HRQoL remained unexplained by the current mediation model. The findings of this study indicate that depression is associated with cardiac anxiety and HRQoL among patients with CVD. In particular, higher levels of depression were associated with increased levels of fear and avoidance, which were subsequently associated with lower HRQoL. These results suggest that avoidance could play an important role in explaining the relationship between depression and HRQoL, highlighting a potential psychological pathway underlying this association.

Although these indirect pathways were statistically significant, their clinical significance should be interpreted with caution. The findings suggest potential targets for psychosocial assessment and nursing intervention, particularly avoidance-related responses; however, the magnitude of the explained variance indicates that additional clinical, behavioral, and contextual factors may also contribute to HRQoL in patients with CVD. Therefore, these results should be understood as identifying statistically significant psychological pathways, rather than as demonstrating clinically sufficient explanations for HRQoL.

## 4. Discussion

This study examined the mediating roles of fear, attention, and avoidance in the relationship between depression and HRQoL. Depression was significantly associated with HRQoL in patients with CVD. Specifically, higher depression levels were associated with lower physical and mental QoL. Depression had a total relationship with HRQoL, as well as a direct relationship, with an average depression score of 5.5 (4.9), rated as mild depression. We designed this study to be exploratory. We wanted to identify potential psychological pathways associated with HRQoL, not establish definitive mechanisms. The model accounted for only 18% of the variance in HRQoL, so our findings make a meaningful but small explanatory contribution to the literature. Other unmeasured variables likely contribute to the remaining variance. Our results align with previous studies reporting that depression is associated with declines in physical and mental health, which relate to lower overall HRQoL.^[31–33]^

A recent study demonstrated that high levels of depression are associated with low HRQoL.^[34]^ Particularly, depressive symptoms correlated significantly with self-reported health status, including symptom burden, physical limitations, overall HRQoL, and general health. More than half of the participants in this study reported moderate to severe physical strain when asked about the physical demands of their work. Moreover, 53.9% of the patients reported no engagement in physical activity, suggesting that the absence of physical activity might contribute to their physical limitations. Thus, depression might be associated with low perceived health and a negative life outlook among patients with CVD. Consequently, routinely assessing depression in patients with CVD could be useful for identifying patients who require additional psychosocial support to improve their HRQoL. Additionally, our results are consistent with those of Lichtman et al^[35]^ who reported that depression is linked to adverse outcomes in CVD, including impaired physical functioning and poor mental health. Furthermore, our findings indicate that depression in patients with CVD is associated with higher-than-average levels of anxiety, including both its physical and psychological aspects.

In patients with CVD, anxiety is associated with an increased severity and frequency of angina-related symptoms and increases the likelihood of death from cardiovascular causes.^[36]^ Specifically, patients with high cardiovascular anxiety are more likely than others to experience intense fear and worry about chest pain or heart-related sensations. Those physical symptoms can be accompanied by fear and concern about death or a heart attack, especially when acute chest pain or changes in heart function occur. Patients with such fears often show heightened attention and vigilance toward their cardiac-related sensations. Anxiety can thus be associated with increased focus and vigilance toward perceived cardiac threats. Although avoidance can sometimes provide temporary relief from unpleasant emotions, it does not offer an effective solution. Consequently, avoidance can be associated with difficulties in treatment engagement and low HRQoL.

Within the construct of anxiety, its subdomains of fear, attention, and avoidance could be differentially associated with depressive symptoms and HRQoL. For instance, fear, defined as intense emotional and physiological responses to perceived threats, is associated with chronic stress and feelings of helplessness, which could increase a person’s vulnerability to depression.^[15]^ Additionally, anxiety-related attentional biases, which are characterized by hypervigilance toward negative or threat-related stimuli, have been discussed as potential cognitive correlates of depressive symptoms.^[37]^ Moreover, avoidance behaviors, which are often used to mitigate fear, can limit exposure to positive experiences and social engagement. Such withdrawal can thus be associated with a small number of opportunities for positive reinforcement and persistent anxiety or depressive symptoms.^[38,39]^ Taken together, the anxiety subdomains could represent interrelated psychological responses rather than a confirmed causal cycle. The findings of this study suggest that the specific and interactive roles of fear, attention, and avoidance provide useful information for developing future targeted psychosocial or nursing interventions, even though their causal and temporal relationships require further investigation.

A statistically significant portion of the variance in HRQoL was explained by the mediating effects of fear, attention, and avoidance, as well as by the overall model, indicating that HRQoL is also influenced by factors beyond the variables included in the model. The mediation analysis showed that indirect effects via the anxiety subdomains accounted for approximately 18% of the variance in HRQoL in the total model. Considering that HRQoL is shaped by myriad factors across various domains of life, this level of explanatory power should be interpreted cautiously rather than as sufficient. A substantial proportion of variance remains unexplained, suggesting that additional factors, such as social, clinical, and environmental variables, might also play important roles in determining HRQoL. In other words, the specific mechanisms of anxiety (fear, attention, and avoidance) might be relevant psychological correlates of patients’ self-evaluation of their HRQoL. Thus, the unique contributions of these anxiety subdomains, in addition to more traditionally studied psychological variables such as coping styles, could help to broaden the understanding of HRQoL in patients with CVD.

Our results show that higher depression in patients with CVD is associated with lower HRQoL, both directly and indirectly, through the mediating effects of the anxiety subdomains of fear, attention, and avoidance. Those mediators might reflect psychological responses that are relevant to how patients perceive and manage distressing health experiences. Consequently, psychological counseling, supportive communication, or nursing interventions are potential strategies to address emotional distress and anxiety-related responses in patients with CVD. In particular, nursing interventions and collaborative efforts between physicians and nurses could focus on helping patients to recognize and manage distressing aspects of their illness experience. Thus, our findings may help generate hypotheses for developing supportive care strategies, such as meaning-centered discussions or coping-focused education; however, their clinical effectiveness would need to be established through future intervention studies before being applied in practice. This integrated approach, rooted in both clinical and nursing practices, could help to improve patient-centered care for individuals with CVD.

Consequently, even though the present findings should be interpreted as preliminary because of the observational design and limited explained variance, targeted patient-centered nursing interventions can be considered to address maladaptive anxiety responses in patients with CVD. For instance, meaning-centered counseling, which assists patients in reconstructing a coherent and meaningful narrative about their experience with illness, has been shown to improve the emotional well-being of individuals with advanced illnesses.^[40]^ In addition, behavioral activation strategies that encourage patients to gradually reengage in pleasurable and meaningful activities can alleviate depressive symptoms and reduce anxiety.^[41]^ Moreover, structured reengagement programs, often led by nurses in interdisciplinary settings, have demonstrated effectiveness in promoting self-care and psychological stability in patients with CVD.^[42]^ Integrating those tailored interventions into routine cardiovascular care could be a promising direction for future intervention studies.

This study has some limitations. First, the cross-sectional study design makes it difficult to establish definitive causal relationships and temporal sequences among depression, anxiety subdomains, and HRQoL. Future studies with longitudinal or experimental designs are required to confirm the directionality of these associations. Second, although the model explains a statistically significant proportion of the variance in HRQoL, it does not capture the full complexity of influencing factors, as variables such as socioeconomic status, social support, personality traits, and comorbidities were not included. Third, the study cohort comprised a specific population of patients with CVD; therefore, the findings might not be directly generalizable to other populations or settings. Fourth, this study excluded participants with a prior diagnosis of depression or anxiety to focus on associations among subclinical symptoms. Although this ensured a homogeneous sample, it might have introduced selection bias, and the findings might not be generalizable to patients with preexisting clinical psychiatric conditions. Fifth, because all the primary variables were assessed using self-reported questionnaires completed by the same participants at a single point in time, the potential for common method bias cannot be entirely ruled out, though we addressed it in our analysis. Finally, despite the use of advanced statistical techniques to control for known confounders, unmeasured or unknown confounding factors might have influenced the observed associations.

In conclusion, this study shows that depression in patients with CVD was significantly associated with lower HRQoL through direct and indirect pathways. Specifically, higher levels of depression were associated with lower physical and psychological HRQoL, and that was partially mediated by the anxiety subdomains of fear, attention, and avoidance. Additionally, increased depression was associated with heightened anxiety and negative health perceptions, suggesting a potential psychological pathway that should be examined further in longitudinal studies. These findings underscore the importance of routine assessments for depression and anxiety in patients with CVD. Moreover, these findings may provide a basis for generating hypotheses regarding targeted, meaning-centered psychological and nursing interventions that focus on the specific roles of fear, attention, and avoidance. However, their effectiveness in improving HRQoL must be confirmed through future intervention studies before clinical application can be recommended. Overall, these findings provide valuable insights into the complex interplay among psychological factors and cardiovascular outcomes, paving the way for refined clinical approaches. Future longitudinal studies are needed to establish temporal relationships and clarify causal pathways among depression, anxiety subdomains, and HRQoL. Furthermore, these results underscore the need for tailored, patient-specific nursing interventions that support the management of depression and anxiety and contribute to improved HRQoL in patients with CVD.

## Acknowledgments

We thank all of the participants and researchers who participated in this study.

## Author contributions

**Conceptualization:** Hwan-Cheol Park, Jihyun Oh.

**Data curation:** Hwan-Cheol Park, Jihyun Oh.

**Investigation:** Hwan-Cheol Park, Jihyun Oh.

**Methodology:** Hwan-Cheol Park, Jihyun Oh.

**Resources:** Hwan-Cheol Park.

**Software:** Hwan-Cheol Park.

**Supervision:** Hwan-Cheol Park.

**Writing – original draft:** Hwan-Cheol Park, Jihyun Oh.

**Formal analysis:** Jihyun Oh.

**Funding acquisition:** Jihyun Oh.

**Project administration:** Jihyun Oh.

**Validation:** Jihyun Oh.

**Visualization:** Jihyun Oh.

**Writing – review & editing:** Jihyun Oh.


