# Supplementary material for: Depression and health-related quality of life in cardiovascular patients: A cross-sectional study on the mediating roles of fear, attention, and avoidance
Source: Medicine (Baltimore). 2026 Aug 7;105(32):e50147. doi: 10.1097/MD.0000000000050147 (PMC13456897; doi:10.1097/MD.0000000000050147)
Supplement: Supplementary file 1 [file medi-105-e50147-s001.docx]

Supplementary Table 1. Sensitivity analyses of the mediation model adjusted for key potential confounders (*N*=204)

| Model | Effect | SE | *t* | *p* | 95% BC CI |
| --- | --- | --- | --- | --- | --- |
| Total effect of depression on quality of life | -.523 | .120 | -4.35 | < .001 | [-.760, -.286] |
| Direct effect of depression on quality of life | -.372 | .120 | -3.094 | .002 | [-.609, -.135] |
| Total indirect effect | -.150 | .069 |  |  | [-.298, -.028] |
| Indirect effect via |  |  |  |  |  |
| Indirect 1 | .018 | .041 |  |  | [-.060, .107] |
| Indirect 2 | -.002 | .016 |  |  | [-.036, .035] |
| Indirect 3 | -.075 | .048 |  |  | [-.185, -.001] |
| Indirect 4 | -.031 | .026 |  |  | [-.091, .012] |
| Indirect 5 | -.027 | .014 |  |  | [-.060, -.003] |
| Indirect 6 | -.002 | .013 |  |  | [-.030, .022] |
| Indirect 7 | -.029 | .014 |  |  | [-.063 -.008] |

Note*.* This mediation model adjusted for age, sex, and clinical comorbidities as covariates. Indirect effects were considered significant when the 95% bias-corrected bootstrap confidence interval did not include zero. BC CI, bias-corrected confidence interval
